# Angiotensin-converting-enzyme inhibitor prevents skeletal muscle fibrosis in myocardial infarction mice

**DOI:** 10.1186/s13395-020-00230-9

**Published:** 2020-04-25

**Authors:** Naoya Kakutani, Shingo Takada, Hideo Nambu, Junichi Matsumoto, Takaaki Furihata, Takashi Yokota, Arata Fukushima, Shintaro Kinugawa

**Affiliations:** 1 Department of Cardiovascular Medicine, Faculty of Medicine and Graduate School of Medicine, Hokkaido University, Kita-15, Nishi-7, Kita-ku, Sapporo, 060-8638 Japan; 2 Japan Society for the Promotion of Science, Tokyo, Japan; 3 Faculty of Lifelong Sport, Department of Sports Education, Hokusho University, Ebetsu, Japan

## Abstract

**Background:**

Transforming growth factor beta (TGF-β)-Smad2/3 is the major signaling pathway of fibrosis, which is characterized by the excessive production and accumulation of extracellular matrix (ECM) components, including collagen. Although the ECM is an essential component of skeletal muscle, fibrosis may be harmful to muscle function. On the other hand, our previous studies have shown that levels of angiotensin II, which acts upstream of TGF-β-Smad2/3 signaling, is increased in mice with myocardial infarction (MI). In this study, we found higher skeletal muscle fibrosis in MI mice compared with control mice, and we investigated the mechanisms involved therein. Moreover, we administered an inhibitor based on the above mechanism and investigated its preventive effects on skeletal muscle fibrosis.

**Methods:**

Male C57BL/6 J mice with MI were created, and sham-operated mice were used as controls. The time course of skeletal muscle fibrosis post-MI was analyzed by picrosirius-red staining (days 1, 3, 7, and 14). Mice were then divided into 3 groups: sham + vehicle (Sham + Veh), MI + Veh, and MI + lisinopril (an angiotensin-converting enzyme [ACE] inhibitor, 20 mg/kg body weight/day in drinking water; MI + Lis). Lis or Veh was administered from immediately after the surgery to 14 days postsurgery.

**Results:**

Skeletal muscle fibrosis was significantly increased in MI mice compared with sham mice from 3 to 14 days postsurgery. Although mortality was lower in the MI + Lis mice than the MI + Veh mice, there was no difference in cardiac function between the 2 groups at 14 days. Skeletal muscle fibrosis and hydroxyproline (a key marker of collagen content) were significantly increased in MI + Veh mice compared with the Sham + Veh mice. Consistent with these results, protein expression of TGF-β and phosphorylated Smad2/3 in the skeletal muscle during the early time points after surgery (days 1–7 postsurgery) and blood angiotensin II at 14 days postsurgery was increased in MI mice compared with sham mice. These impairments were improved in MI + Lis mice, without any effects on spontaneous physical activity, muscle strength, muscle weight, and blood pressure.

**Conclusions:**

ACE inhibitor administration prevents increased skeletal muscle fibrosis during the early phase after MI. Our findings indicate a new therapeutic target for ameliorating skeletal muscle abnormalities in heart diseases.

## Background

Fibrosis is associated with an impairment of various organs, including the heart, kidney, lung, and skeletal muscle, and has become a major cause of death in the developed world [1, 2]. Recently, skeletal muscle fibrosis has been reported to be caused by several diseases (e.g., chronic kidney disease and Duchenne muscular dystrophy) [3, 4], or in response to injury [5] and immobilization [6]. Although the extracellular matrix (ECM) is an essential component of skeletal muscle, skeletal muscle fibrosis is characterized by the excessive production and accumulation of collagen and other ECM components, resulting in cellular dysfunction and the loss of tissue architecture, eventually leading to organ failure [7].

Skeletal muscle abnormalities leading to exercise intolerance is a major determinant of the prognosis, overall health, activities-of-daily living, and quality-of-life of patients with heart failure (HF) [8–10]. Our group has been studying qualitative abnormalities in the skeletal muscle of patients with HF after myocardial infarction (MI) [11–13] and in patients with HF [14–16]. However, the presence and mechanism of skeletal muscle fibrosis remains unclear.

The molecular pathways involved in fibrosis are well-known and common among some organs (e.g., the heart and kidney). Transforming growth factor beta (TGF-β) is one of the main signaling molecules initiating fibrosis [17]. Active TGF-β binds to the TGF-β receptor and induces the phosphorylation and activation of Smad2/3 transcription factors [18, 19]. On the other hand, one of the multiple functions of the renin-angiotensin system (RAS) is known to be the activation of TGF-β [20, 21]. We and another group previously found that RAS is activated in HF mice after MI [22, 23] as well as in patients with HF [24, 25]. However, it is unclear whether skeletal muscle fibrosis is induced by the induction of MI by surgery.

Therefore, in this study, we first sought to determine whether skeletal muscle fibrosis is present in mice with MI. We further analyzed whether skeletal muscle fibrosis in MI mice can be prevented by administration of an inhibitor of angiotensin-converting enzyme (ACE).

## Methods

### Experimental animals

Male C57BL/6 J mice (10–12 weeks old, body weight [BW] 23–25 g, SLC Japan, Shizuoka, Japan) were bred in a pathogen-free environment and housed in an animal room under controlled conditions on a 12-h light/dark cycle maintained at 23–25 °C. Normal food (CE-2, CLEA, Tokyo, Japan) and water were provided ad libitum.

### Protocol 1: time course of the study

Mice underwent surgery to ligate the left coronary artery (MI, *n* = 80) as described previously [26, 27]. A sham operation without ligation of the coronary artery was also performed (*n* = 6). For both operations, mice were anesthetized with pentobarbital (50 μg/g BW, intraperitoneal administration [i.p.]), and the adequacy of the anesthesia was monitored based on the disappearance of the pedal withdrawal reflex. We used 6 Sham and 68 MI mice excluding intraoperative death mice (*n* = 12). Sham mice were evaluated at day 14 postsurgery. MI mice were randomly assigned to 4 groups for sacrifice on days 1 (*n* = 6), day 3 (*n* = 12), day 7 (*n* = 25), and day 14 (*n* = 25) based on expected survival rates, and we evaluated a total of 24 MI mice (*n* = 6 each) that survived. Assignment of mice to each group was performed just after the surgery, using numerical codes to identify the animals. Echocardiography measurements were performed 1, 3, 7, and 14 days postsurgery, and then, mice were sacrificed, and their skeletal muscles were excised. Histological analyses were subsequently performed.

### Protocol 2: administration of lisinopril

We next analyzed whether an ACE inhibitor, which has been shown to exert secondary anti-fibrotic effects, prevents the increase in skeletal muscle fibrosis of MI mice. First, we examined the effects of administering vehicle (Sham + Veh) or lisinopril (Lis, Cayman Chemical, Ann Arbor, MI, USA) (Sham + Lis) on skeletal muscle fibrosis in Sham mice as a preliminary study. The dose of Lis was chosen on the basis of a previous study (20 mg/kg BW per day for 2 weeks) [28]. As a result, there was no difference in skeletal muscle fibrosis area between the Sham + Veh and Sham + Lis mice (Sham 3.9% ± 0.3% vs. Sham + Lis 3.4% ± 0.3%, *n* = 5, respectively) at 14 days postsurgery. Hence, we performed the subsequent experiments on all mice except for the Sham + Lis group.

Second, to clarify the effects of Lis on skeletal muscle fibrosis in MI mice, Sham mice (*n* = 24) were randomly assigned to 4 groups after the surgery for sacrifice on days 1, 3, 7, and 14 (*n* = 6 each). The MI mice (*n* = 52) were randomly divided into 2 groups after the surgery for treatment with Lis for 2 weeks (MI + Lis, *n* = 29) or without Lis treatment (MI + Veh, *n* = 23) (Supplemental Figure 1). The MI + Veh mice were sacrificed on day 14. The skeletal muscle samples used for immunoblotting of MI + Veh mice in days 1, 3, and 7 were used from those obtained in Protocol 1. The MI + Lis mice were additionally randomly assigned to 4 groups for sacrifice on day 1 (*n* = 6), day 3 (*n* = 7), day 7 (*n* = 8), and day 14 (*n* = 8) based on expected survival rates. The Sham + Veh and the MI + Lis mice were analyzed by echocardiography at 1, 3, 7, and 14 days postsurgery, and then, they were sacrificed, and their skeletal muscles were excised (*n* = 6 each). Blood pressure was noninvasively measured in mice at 14 days postsurgery, and grip strength and spontaneous physical activity were analyzed before sacrifice. Biochemical measurements, immunoblotting, and histological analyses, were subsequently performed.

### Echocardiographic and hemodynamic measurements

Echocardiographic and hemodynamic measurements were performed after the 2-week treatment under light anesthesia with tribromoethanol/amylene hydrate (Avertin, 200 mg/kg BW, i.p.), which is reported to have a short duration of action and modest cardiodepressive effects, and to enable spontaneous respiration [23]. A commercially available focused 13-MHz linear array transducer at a depth setting of 2.0 cm was used. Two-dimensional parasternal short-axis views were obtained at the level of the papillary muscles. In general, the best views were obtained with the transducer lightly applied to the mid-upper left anterior chest wall. The transducer was then gently moved in the cephalad or caudad direction and angulated until desirable images were obtained. Two-dimensional targeted M-mode tracings were recorded at a paper speed of 50 mm/s.

Systolic blood pressure was measured using the tail-cuff method (BP-98A; Softron, Tokyo, Japan) without anesthesia.

### Spontaneous physical activity

Spontaneous physical activity was measured using an animal movement analysis system (ACTIMO System; Shintechno, Fukuoka, Japan), which consists of a rectangular enclosure (30 × 20 cm), with side walls equipped with photosensors at 2-cm intervals. Pairs of photosensors scan animal movement at 0.5-s intervals. Spontaneous activity was recorded in all groups of mice. Mice were placed in individual chambers and housed in cages for 1 day before each recording, to become familiarized with the recording environment. Movement signal count recordings were performed using the Spike 2 analysis program [29].

### Measurement of fibrotic area and cross-sectional area in skeletal muscle

Gastrocnemius muscles were harvested and immediately embedded in OCT compound (Sakura Finetek, Tokyo, Japan) and then frozen in melting isopentane precooled in liquid nitrogen. All samples were stored at − 80 °C until use. Frozen gastrocnemius muscles were cut into 10-μm-thick sections using a Microm HM 550 cryostat (Microm, Walldorf, Germany). Skeletal muscle fibrosis and cross-sectional area (CSA) were histologically assessed by picrosirius red and hematoxylin and eosin staining, respectively, as previously described [30, 31]. Images at × 40 and × 20 magnification were taken using a BZ-X710 microscope (KEYENCE, Osaka, Japan). Morphological analysis was performed using the BZ-X Analyzer software (KEYENCE). Fibrotic area was analyzed in nine sections per mice. Threshold analysis was carried out on each section to quantify the area occupied by collagen deposits as a percentage of the total area. A mean value was generated based on the results from the nine sections. The measurements of CSA were performed in at least 100 cells from each mouse, and the average was reported.

### Measurement of hydroxyproline

Hydroxyproline is a major component of protein collagen. The hydroxyproline content of the soleus muscle of mice at 14 days postsurgery was determined using the Hydroxyproline Assay Kit (QuickZyme Biosciences, Leiden, Netherlands), according to the manufacturer’s instructions.

### Measurement of circulating angiotensin II

Serum angiotensin II levels were measured using an enzyme immunoassay kit (Phoenix Pharmaceuticals, CA, USA).

### Immunoblotting

Immunoblotting was performed as described previously [29]. Briefly, hindlimb skeletal muscle tissue samples were homogenized in 1 × cell lysis buffer (Cell Signaling Technology, MA, USA) supplemented with 1 × Complete Protease Inhibitor Cocktail (Roche, Basel, Switzerland), 1 × Phosphatase Inhibitor Cocktail 2 (Sigma, MO, USA), Phosphatase Inhibitor Cocktail 3 (Sigma, MO, USA), and 1 mmol/L phenylmethylsulfonyl fluoride. After sonification and centrifugation at 15,000 *g* for 10 min at 4 °C, the supernatants were collected. Protein aliquots were taken for the total protein assay (Pierce BCA, IL, USA), and the remaining lysate (20 μg) was added loaded onto 10% polyacrylamide gels (Bio-Rad, CA, USA), electrophoretically separated by sodium dodecyl sulfate-polyacrylamide gel electrophoresis (SDS-PAGE) and transferred by electroblotting to a polyvinylidene fluoride membrane (Bio-Rad) using transfer buffer at 20 V, overnight.

After blocking in tris-buffered saline with 0.1% Tween-20 (TBS-T) in 5% non-fat dry milk or 5% albumin, the membranes were incubated overnight at 4 °C with primary antibodies against collagen I (Abcam, Cambridge, UK), TGF-β (Abcam), Smad2/3 (Cell Signaling Technology, Danvers, USA), phosphorylated forms of Smad2/3 (p-Smad2/3) (Cell Signaling Technology, Danvers, USA), p44/42 mitogen-activated protein kinase (MAPK) (Cell Signaling Technology, Danvers, USA), and phosphorylated forms of p44/42 MAPK (p-MAPK) (Cell Signaling Technology, Danvers, USA) (all at a dilution of 1:1000). After being washed three times in TBS-T buffer, the membranes were incubated with secondary antibodies conjugated with horseradish peroxidase (dilution 1:50,000; Abcam). The membranes were washed again in TBS-T and incubated with the chemiluminescence detection reagent, ECL^TM^ or ECL prime Western Blotting Analysis System (GE Healthcare, Amersham, UK) to enhance their chemiluminescence. The bands were visualized by enhanced chemiluminescence and quantified using the Image J software (National Institutes of Health, MD, USA) [32]. The resulting values were expressed as the ratio of target band intensity to total protein, evaluated by Coomassie brilliant blue (CBB) staining. CBB staining was used as an internal control to normalize the results and to control for blot-to-blot variation.

### Statistical analysis

Data are expressed as the mean ± standard error (SE). Comparisons among groups were performed using the paired *t* test, one-way ANOVA, and chi-square test. Post hoc comparisons were made by the Dunnett or Tukey test. Kaplan-Meier analysis with the log-rank test was performed to compare the survival rates among 3 groups for 14 days postsurgery. A *P* value of less than 0.05 was considered to indicate a statistically significant difference between the 2 groups. All statistical analyses were performed using Prism Version 8 (Graph Pad Software, Inc., CA, USA).

## Results

### Time course of skeletal muscle fibrosis after MI

**Fig. 1:**
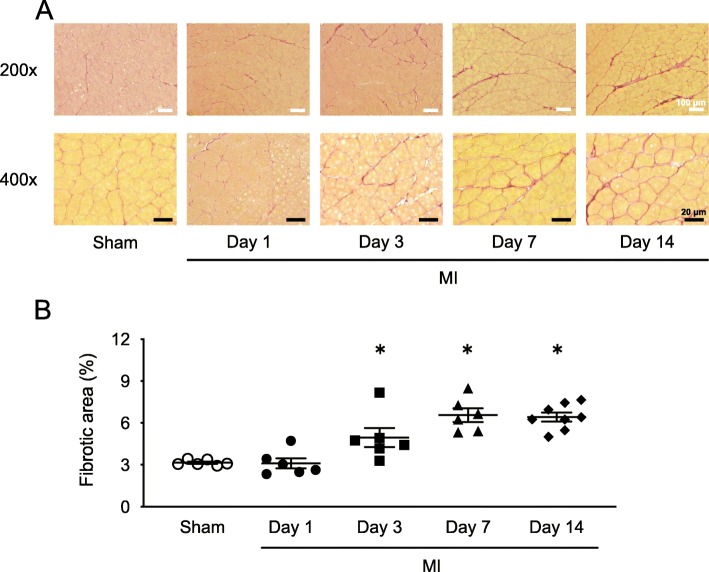
Time course of skeletal muscle fibrosis exacerbation in MI mice. **a** Representative photomicrographs of picrosirius red-stained gastrocnemius cross-sections from Sham and MI mice at 1, 3, 7, and 14 days postsurgery. *Top* panels show low magnification micrographs (× 200; white scale bars = 100 μm), and *bottom* panels show higher magnification micrographs (× 400; black scale bars = 20 μm). **b** Summary data of fibrotic areas. Data are expressed as the mean ± SE (*n* = 6); *P < 0.05 vs. Sham

We first sought to determine whether skeletal muscle fibrosis is present in MI mice. Figure 1 a shows representative high-power photomicrographs of gastrocnemius cross-sections stained with picrosirius red. Figure 1 b shows the summarized data of morphological analysis in sham mice and MI mice at 1, 3, 7, and 14 days postsurgery. Skeletal muscle fibrosis was significantly increased in the MI mice from 3 to 14 days postsurgery compared with sham mice. Therefore, these results indicated that treatment from an early phase post-MI would be necessary to suppress the increase in skeletal muscle fibrosis.

### Effects of Lis on animal characteristics

**Fig. 2:**
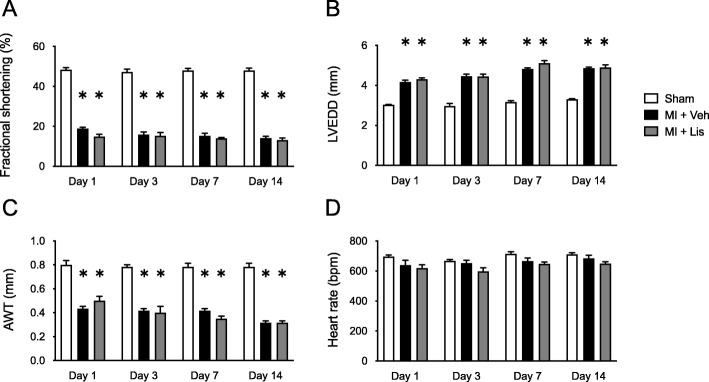
Time course of echocardiography parameters in Sham + Veh, MI + Veh, and MI + Lis mice. Summarized data of fractional shortening (**a**), left ventricular end diastolic diameter (LVEDD) (**b**), anterior wall thickness (AWT) (**c**), and heart rate (**d**). Data are expressed as the mean ± SE (*n* = 6); *P < 0.05 vs. Sham; MI, myocardial infarction; Veh, vehicle; Lis, lisinopril

Table 1 shows the characteristics of the Sham, MI, and MI + Lis mice at 14 days postsurgery. BW, lung weight/BW, and gastrocnemius muscle weight/BW showed no differences among the three groups. Heart weight/BW was significantly increased in the MI and MI + Lis mice compared with the sham mice. LV end-diastolic diameter was significantly increased, and fractional shortening and anterior wall thickness (AWT) were significantly decreased in MI + Veh and MI + Lis mice compared with Sham mice, but there was no difference in the function of and the AWT of the LV between the MI groups. Systolic blood pressure was significantly decreased in MI + Lis mice compared with sham mice (Table 1). We measured cardiac function by echocardiography in the 3 groups at 1, 3, 7, and 14 days postsurgery. Left ventricle (LV) end-diastolic diameter was significantly increased, and fractional shortening and anterior wall thickness (AWT) of the LV were significantly decreased in MI + Veh and MI + Lis mice compared with Sham + Veh mice at 1, 3, 7, and 14 days postsurgery, but there was no difference in the function and AWT of the LV between the MI groups (Fig. 2). In addition, there was no difference in heart rate among the 3 groups (Fig. 2). Serum Ang II level was significantly increased in MI mice compared with sham mice and was significantly decreased in MI + Lis mice compared with MI mice (Table 1). From these results, we confirmed the effect of Lis on MI mice. On the other hand, there were no significant differences in the CSA of gastrocnemius muscle, grip strength, and spontaneous physical activity among the three groups (Table 1). In the MI group, the survival rate was significantly higher in MI + Lis mice than in the MI + Veh group at 14 days postsurgery (26% [MI + Veh mice] vs. 75% [MI + Lis mice]) (Supplemental Figure 2). It is an established fact that ACE inhibitor administration significantly improves the mortality of a post-MI rodent model [33], and hence, this may be occurring in our study. However, we did not investigate the cause of death of the mice in each MI group.

**Table 1:** Characteristics of the mice in Protocol 2

	Sham + Veh	MI + Veh	MI + Lis
Total number	6	6	6
Body and organ weight			
BW, g	24.5 ± 0.3	25.0 ± 0.4	24.3 ±0.6
Heart weight/BW, mg/g	4.4 ± 0.2	5.8 ± 0.3*	5.8 ± 0.3*
Lung weight/BW, mg/g	5.5 ± 0.1	7.1 ± 0.7	6.4 ± 0.5
Gastrocnemius weight/BW, mg/g	5.6 ± 0.1	5.4 ± 0.1	5.4 ± 0.1
Noninvasive blood pressure measurements			
SBP, mmHg	99 ± 2	95 ± 2	89 ± 3*
Serum angiotensin II, ng/mL	10.9 ± 2.0	26.2 ± 5.1*	4.0 ± 1.5*†
Skeletal muscle cross-sectional area, μm^2^	2269 ± 141	2028 ± 21	2100 ± 94
Grip strength, N	1.52 ± 0.03	1.53 ±0.04	1.58 ± 0.07
Spontaneous physical activity, count/day	2104 ± 416	1916 ± 410	2350 ± 281

Data are expressed as the mean ± S.E.M. *n* = 6

*BW* body weight, *DBP* diastolic blood pressure, *Lis* lisinopril, *MI* myocardial infarction, *SBP* systolic blood pressure, *Veh* vehicle

*P < 0.05 vs. Sham + Veh, †*P* < 0.05 vs. MI + Veh

We next analyzed whether an ACE inhibitor, which has been shown to exert secondary anti-fibrotic effects [34], prevents the increase in skeletal muscle fibrosis of MI mice.

### Effects of Lis on skeletal muscle fibrosis

**Fig. 3:**
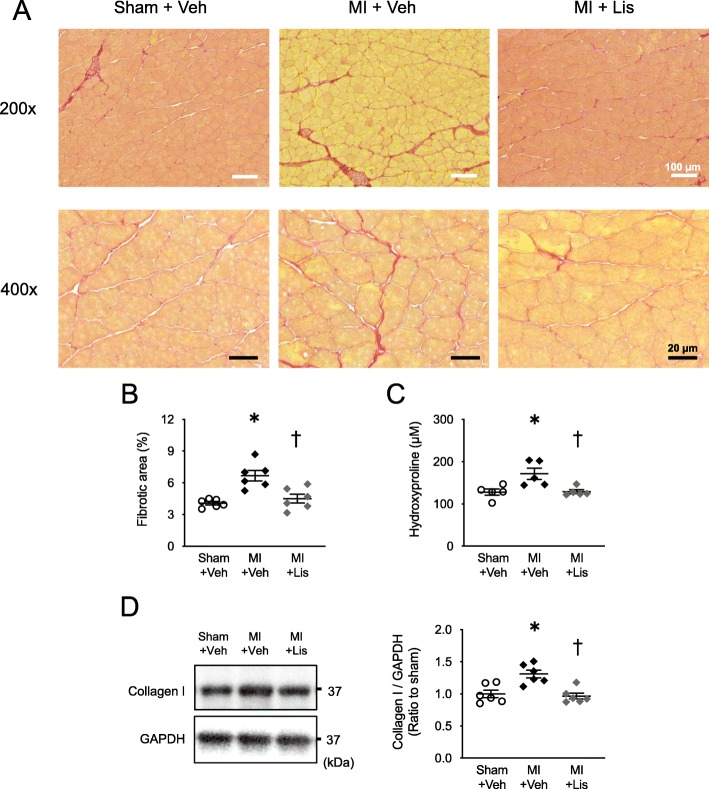
Effects of Lis administration on skeletal muscle fibrosis in MI mice. **a** Representative high-power photomicrographs of gastrocnemius cross-sections stained with picrosirius red from Sham, MI, and MI + Lis mice at 14 days postsurgery. *Top* panels show low magnification micrographs (× 200; white scale bars = 100 μm), and *bottom* panels show higher magnification micrographs (× 400; black scale bars = 20 μm). **b** Summary data of fibrotic area (*n* = 6). **c** Quantification of hydroxyproline levels in soleus muscle from 3 groups at 14 days postsurgery (*n* = 5). **d** Representative data of immunoblotting of gastrocnemius muscle lysates obtained from the 3 groups (*left*). Right: summarized data of collagen protein expression level (*n* = 6). GAPDH (glyceraldehyde-3-phosphate dehydrogenase) was used as an internal control. The sizes of the molecular weight markers are indicated on the right in kilo Dalton. Data are expressed as the mean ± SE. *P < 0.05 vs. Sham + Veh, ^†^*P* < 0.05 vs. MI + Veh

Figure 3 a shows representative images of picrosirius red staining. Fibrotic area, skeletal muscle hydroxyproline level (a key marker of collagen content), and collagen I protein level were significantly increased in MI mice compared with sham mice and were at normal levels in the MI + Lis mice (Fig. 3b, c).

**Fig. 4:**
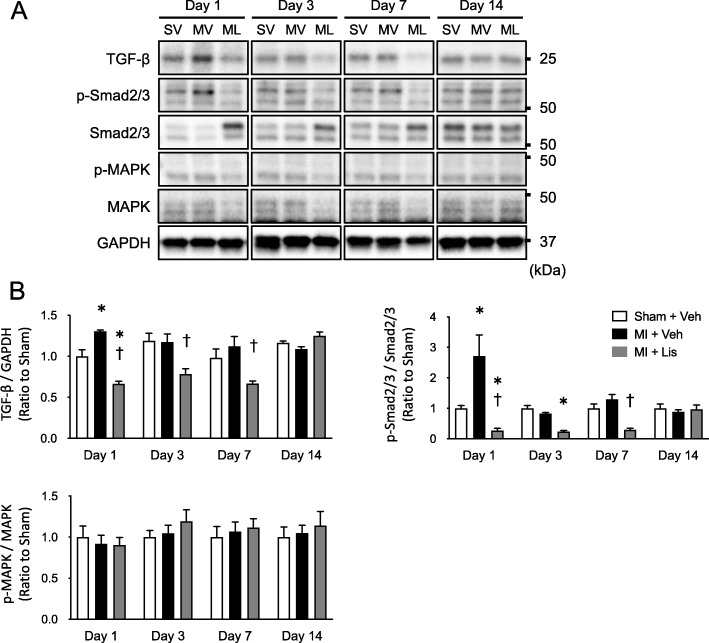
Signal pathway inducing skeletal muscle fibrosis after MI. **a** Representative immunoblotting bands in gastrocnemius muscle obtained from the 3 groups. The sizes of the molecular weight markers are indicated on the right in kilo Dalton. **b** Summarized data of protein expression of TGF-β, p-Smad/Smad, and p-MAPK/MAPK. GAPDH was used as an internal control. Data are expressed as the mean ± SE. *P < 0.05 vs. Sham + Veh, ^†^*P* < 0.05 vs. MI + Veh. TGF-β, transforming growth factor beta; MAPK, mitogen-activated protein kinase. Data are expressed as the mean ± SE. *P < 0.05 vs. Sham, †*P* < 0.05 vs. MI. SV, Sham + Vehicle; MV, MI + Vehicle, ML, MI + Lisinopril; TGF-β, transforming growth factor beta; MAPK, mitogen-activated protein kinase

Protein levels of TGF-β and phosphorylated Smad2/3 per total Smad in the early phase were significantly increased in MI mice compared with sham mice and were significantly suppressed in the MI + Lis mice (Fig. 4b). These changes were not observed in phosphorylated p44/42 MAPK/total MAPK (Fig. 4b), which are components of an important signaling pathway for ECM production [35].

## Discussion

To investigate whether skeletal muscle fibrosis is increased after MI, we first analyzed the time course of skeletal muscle fibrosis after MI. Skeletal muscle fibrosis significantly increased from day 3 to day 14 postsurgery in MI mice compared with sham mice (Fig. 1). We next analyzed whether a mouse model with MI-induced skeletal muscle fibrosis improves by administration of an ACE inhibitor. Lis administration significantly inhibited skeletal muscle fibrosis in MI mice without affecting organ weights, muscle CSA, LV function, muscle strength, and spontaneous physical activity (Table 1). Consistent with these results, the levels of blood angiotensin II and TGF-β-Smad2/3 signaling in the skeletal muscle were also decreased to normal levels by Lis in MI mice (Table 1 and Fig. 3).

### Skeletal muscle fibrosis in heart diseases and its possible mechanism

Previous studies have mainly reported that skeletal muscle fibrosis is induced by gene mutations [5], mechanical and chemical injury [35], and fixed inactivity [36]. We showed that skeletal muscle fibrosis and the level of hydroxyproline, a key marker of collagen content, were increased in MI model mice (Fig. 3). Similar to our results, skeletal muscle fibrosis was observed in young patients with heart disease [37], and cardiac hypertrophy was observed in spontaneous hypertensive rats [38], but the underlying mechanism remains unclear. On the other hand, our established MI model mice did not show changes in blood pressure compared with sham or MI + Lis mice (Table 1) [13, 39]. On the other hand, we previously found that serum angiotensin II levels were increased in MI mice [22, 23], as well as in many clinical studies [24, 25]. One of the multiple functions of RAS activation is known to be the enhancement of TGF-β signaling, and the molecular pathways involved in fibrosis are well-known and common in some organs. TGF-β is one of the main signaling molecules initiating fibrosis [17]. Consistent with the results of studies focusing on myocardial fibrosis, serum Ang II level in MI mice was increased in the present study (Table 1). Moreover, protein expression of TGF-β and phosphorylated Smad2/3, downstream signaling molecules of RAS were also increased in skeletal muscle (Fig. 4). Therefore, we next analyzed the effects of treatment of MI mice with the RAS inhibitor Lis. As a result of treatment with Lis, an increase in skeletal muscle fibrosis, as well as serum Ang II and TGF-β-Smad2/3 signaling in the skeletal muscle, was also prevented in the skeletal muscle of MI mice without affecting cardiac function, spontaneous physical activity, and blood pressure (Figs. 3, 4, and Table 1). Therefore, we showed for the first time the possibility of a treatment for skeletal muscle fibrosis associated with a medical disease. However, we previously suggested that skeletal muscle fibrosis does not increase in Ang II-infusion model mice [40]. In other words, our series of results suggest that factors other than Ang II may also be important for the increase of skeletal muscle fibrosis in MI model mice, but this could not be clarified in the present study.

### Time course of skeletal muscle fibrosis in heart disease

Previous studies focusing on skeletal muscle fibrosis have only performed analyses at one timepoint [3]. Therefore, we clarified the time course of skeletal muscle fibrosis after MI, namely at days 1, 3, 7, and 14 postsurgery. Dean et al. observed that collagen protein expression in the border zone of cardiac muscle was significantly increased from day 3 after MI surgery [41]. Similarly, TGF-β protein level was increased only on day 1 after surgery prior to collagen protein expression. The results of these time course analyses appeared to be consistent with an increase in skeletal muscle fibrosis and TGF-β-Smad2/3 signaling of MI mice (Figs. 1 and 4). From these results, it is possible to consider that neurohumoral factors in the blood, which can commonly affect skeletal muscle and cardiac muscle, are playing a role in muscle fibrosis. Therefore, it is suggested that Ang II might play a role in the increase of skeletal muscle fibrosis (Table 1). As our results indicate, skeletal muscle fibrosis occurs mainly during the early phase after post-MI (Fig. 1). Thus, to our knowledge, there is no treatment for fibrosis to date, but preventive or early administration of an ACE inhibitor in MI mice may suppress the increase in skeletal muscle fibrosis [42–44]. These findings are consistent with the clinical guidelines that recommend early administration of ACE inhibitors after MI [45, 46].

### Clinical implications and perspectives

The ECM forms up to 10% of the skeletal muscle weight [47], and it plays an essential role in force transmission and the maintenance and repair of muscle fibers after injury [48]. However, the excessive production and accumulation of ECM components, i.e., fibrosis, induces contractile dysfunction and stiffness in the heart [49]. Recently, Abramowitz et al. reported that skeletal muscle fibrosis was increased in patients with chronic kidney disease and negatively correlates with physical fitness levels [50]. Therefore, there may be an association between skeletal muscle fibrosis and physical fitness levels in heart disease patients. However, there was no association between the above 2 factors. Skeletal muscle fibrosis has been reported to be caused by chronic kidney disease and Duchenne muscular dystrophy, as well as in response to injury and immobilization. The results of skeletal muscle fibrosis in the above study (percentage collagen area by picrosirius red staining: normal group, approximately 8–15% vs. disease groups, approximately 10–35%) are very different from our data in this study (sham group, approximately 3% vs. MI group, 3–8%). Therefore, it is possible that relatively mild skeletal muscle fibrosis occurs in our model, which is not a chronic disease or injury model. Moreover, previous studies reported that excessive collagen accumulation in skeletal muscle correlates with muscle pain and muscle stiffness [51, 52]. We believe further clarification of these points is necessary in the future. On the other hand, a previous study reported that knee extension strength and walking speed was maintained in a patient with hypertension, upon treatment with an ACE inhibitor [53]. Thus, the prevention of skeletal muscle fibrosis using early and continuous ACE inhibitor treatment in heart disease patients may play a role in muscle function.

## Conclusions

ACE inhibitor administration prevents the increase in skeletal muscle fibrosis during the early phase after MI. Our findings may provide a new therapeutic target to skeletal muscle abnormalities in heart diseases. Future studies are required to clarify whether skeletal muscle fibrosis is also directly linked to physical fitness.

## Supplementary information

**Additional file 1: Figure S1.** Study design for protocol 1 and 2. In **Protocol 1**, 6 Sham and 68 MI mice were used. Sham mice were evaluated at day 14 postsurgery. MI mice were randomly assigned to 4 groups after the surgery for sacrifice on day 1 (*n* = 6), day 3 (*n* = 12), day 7 (*n* = 25), and day 14 (*n* = 25), and we evaluated a total of 24 MI mice (*n* = 6 each) that survived. In **Protocol 2**, the Sham mice (*n* = 24) were randomly assigned after surgery for sacrifice on day 1, 3, 7, 14 (*n* = 6 each). The MI mice (*n* = 52) were randomly divided into 2 groups just after the surgery for treatment with Lis for 2 weeks (MI + Lis, *n* = 29), or without Lis treatment (MI + Veh, *n* = 23). The MI + Veh mice were sacrificed on day 14. The MI + Lis mice were randomly assigned to 4 more groups for sacrifice on day 1 (*n* = 6), day 3 (*n* = 7), day 7 (*n* = 8), and day 14 (*n* = 8), and we evaluated a total of 24 MI + Lis mice (*n* = 6 each). MI, myocardial infarction; Veh, vehicle; Lis, lisinopril. **Figure S2.** Survival curves of Sham +Veh, MI + Veh, and MI + Lis mice at 14 days post-surgery. MI, myocardial infarction; Veh, vehicle; Lis, lisinopril (*n* = 6–23).

## Data Availability

The datasets generated or analyzed during the current study are available from the corresponding author upon reasonable request.

## Abbreviations

ECM: Extracellular matrix
HF: Heart failure
MI: Myocardial infarction
TGF-β: Transforming growth factor beta
RAS: Renin-angiotensin system
ACE: Angiotensin-converting enzyme
BW: Body weight
CSA: Cross-sectional area
MAPK: Mitogen-activated protein kinase
