# Supplementary material for: Angiotensin-converting-enzyme inhibitor prevents skeletal muscle fibrosis in myocardial infarction mice
Source: Skelet Muscle. 2020 Apr 25;10:11. doi: 10.1186/s13395-020-00230-9 (PMC7183133; doi:10.1186/s13395-020-00230-9)
Supplement: Supplementary file 1 — Additional file 1: Figure S1. Study design for protocol 1 and 2. In Protocol 1, 6 Sham and 68 MI mice were used. Sham mice were evaluated at day 14 postsurgery. MI mice were randomly assigned to 4 groups after the surgery for sacrifice on day 1 (n = 6), day 3 (n = 12), day 7 (n = 25), and day 14 (n = 25), and we evaluated a total of 24 MI mice (n = 6 each) that survived. In Protocol 2, the Sham mice (n = 24) were randomly assigned after surgery for sacrifice on day 1, 3, 7, 14 (n = 6 each). The MI mice (n = 52) were randomly divided into 2 groups just after the surgery for treatment with Lis for 2 weeks (MI + Lis, n = 29), or without Lis treatment (MI + Veh, n = 23). The MI + Veh mice were sacrificed on day 14. The MI + Lis mice were randomly assigned to 4 more groups for sacrifice on day 1 (n = 6), day 3 (n = 7), day 7 (n = 8), and day 14 (n = 8), and we evaluated a total of 24 MI + Lis mice (n = 6 each). MI, myocardial infarction; Veh, vehicle; Lis, lisinopril. Figure S2. Survival curves of Sham +Veh, MI + Veh, and MI + Lis mice at 14 days post-surgery. MI, myocardial infarction; Veh, vehicle; Lis, lisinopril (n = 6–23). [file 13395_2020_230_MOESM1_ESM.pptx]

## Slide 1
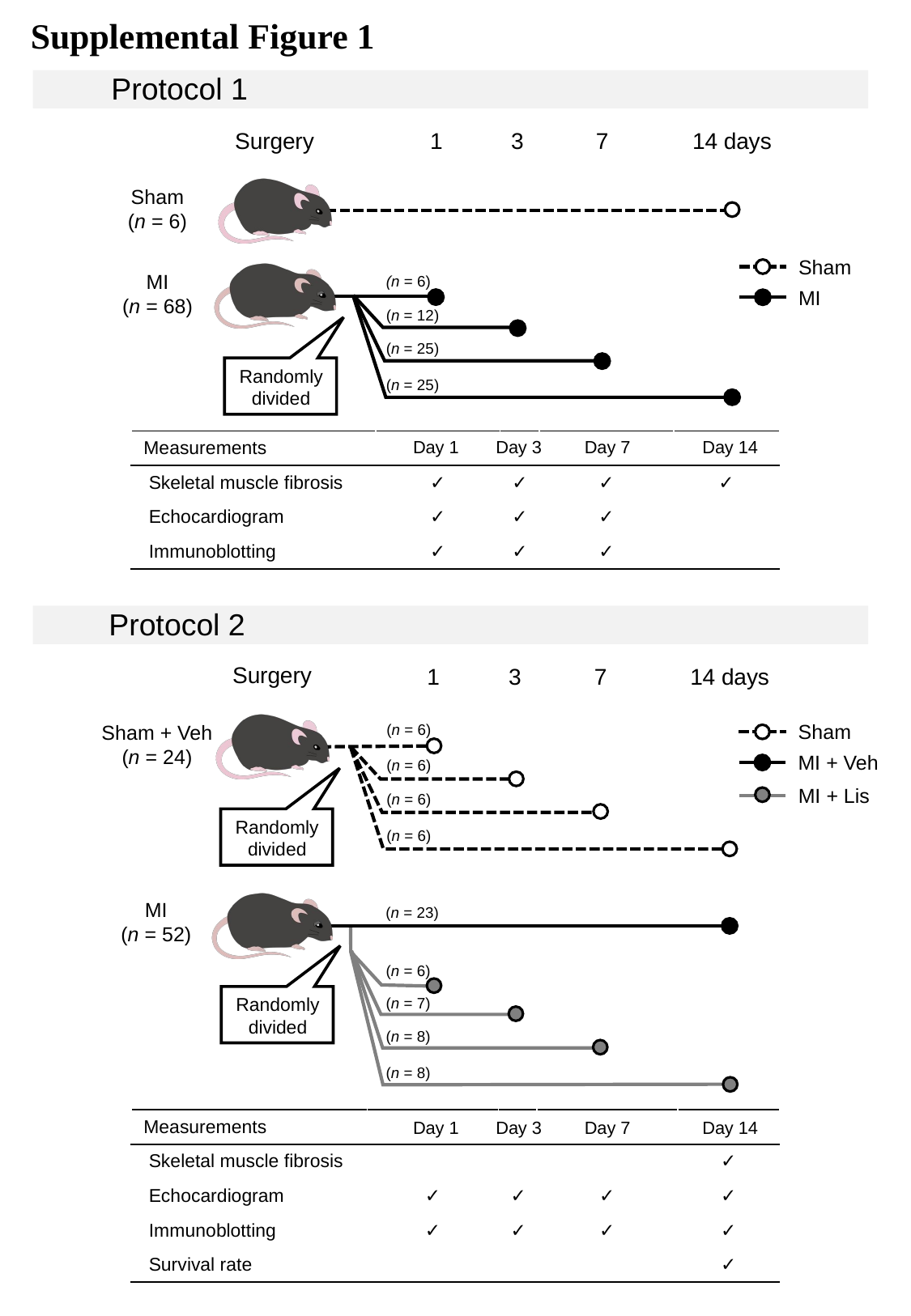

Supplemental Figure 1
Protocol 1
14 days
Surgery
1
3
7
Sham
(n = 6)
MI
(n = 68)
Randomly
divided
Sham
MI
(n = 6)
(n = 12)
(n = 25)
(n = 25)
Day 1
Day 3
Day 7
Day 14
| Measurements | | | | |
| --- | --- | --- | --- | --- |
| Skeletal muscle fibrosis | ✓ | ✓ | ✓ | ✓ |
| Echocardiogram | ✓ | ✓ | ✓ | |
| Immunoblotting | ✓ | ✓ | ✓ | |
Protocol 2
Surgery
14 days
1
3
7
Sham
MI + Veh
MI + Lis
Sham + Veh
(n = 24)
(n = 6)
(n = 6)
(n = 6)
Randomly
divided
(n = 6)
MI
(n = 52)
(n = 23)
(n = 6)
Randomly
divided
(n = 7)
(n = 8)
(n = 8)
| Measurements | | | | |
| --- | --- | --- | --- | --- |
| Skeletal muscle fibrosis | | | | ✓ |
| Echocardiogram | ✓ | ✓ | ✓ | ✓ |
| Immunoblotting | ✓ | ✓ | ✓ | ✓ |
| Survival rate | | | | ✓ |
Day 1
Day 3
Day 7
Day 14

## Slide 2
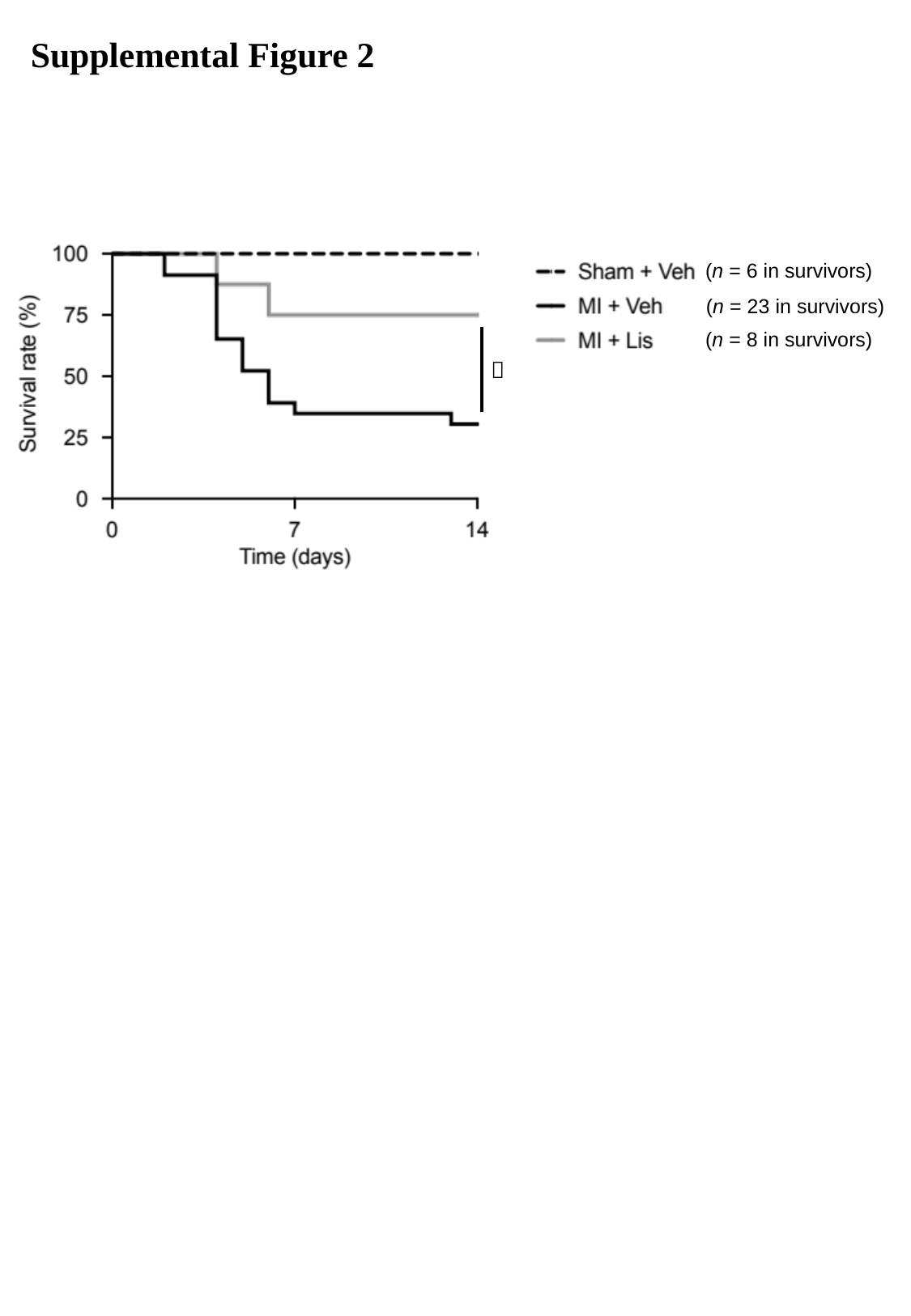

Supplemental Figure 2
(n = 6 in survivors)
(n = 23 in survivors)
(n = 8 in survivors)
＊
